# Supplementary figures and images for: Exploiting embryonic niche conditions to grow Wilms tumor blastema in culture
Source: Front Oncol. 2023 Mar 16;13:1091274. doi: 10.3389/fonc.2023.1091274 (PMC10061139; doi:10.3389/fonc.2023.1091274)

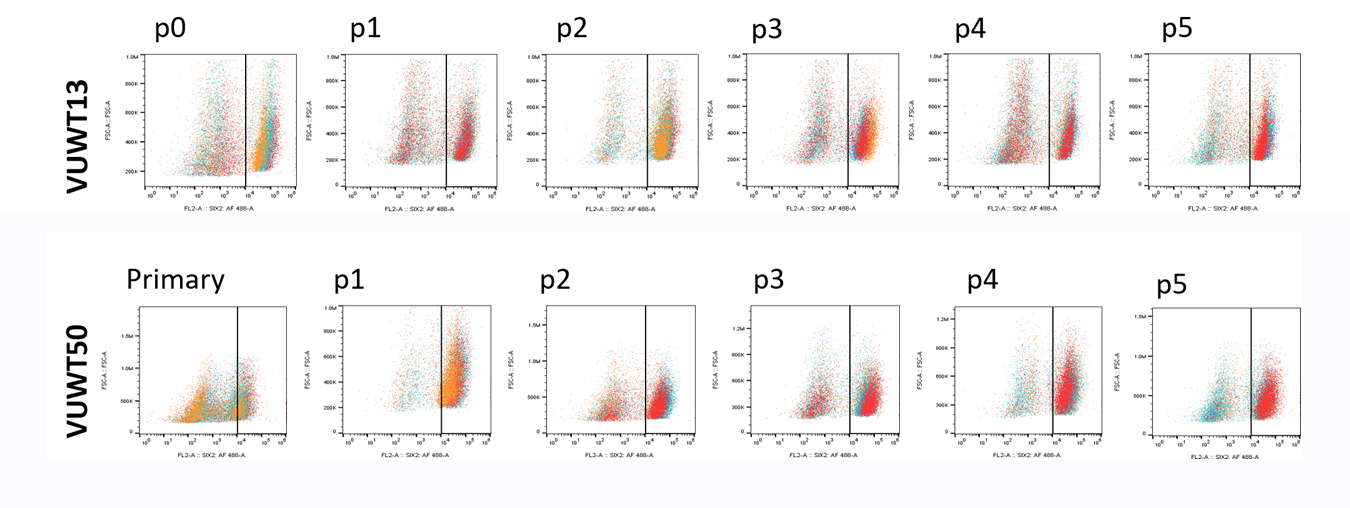

Supplement: Supplementary Figure 1 — Scatter plots of flow cytometric quantification for SIX2+ VUWT13 and VUWT50 cultured and passaged cells. Controls included only secondary antibody for proper gating. [file Image_1.tif]

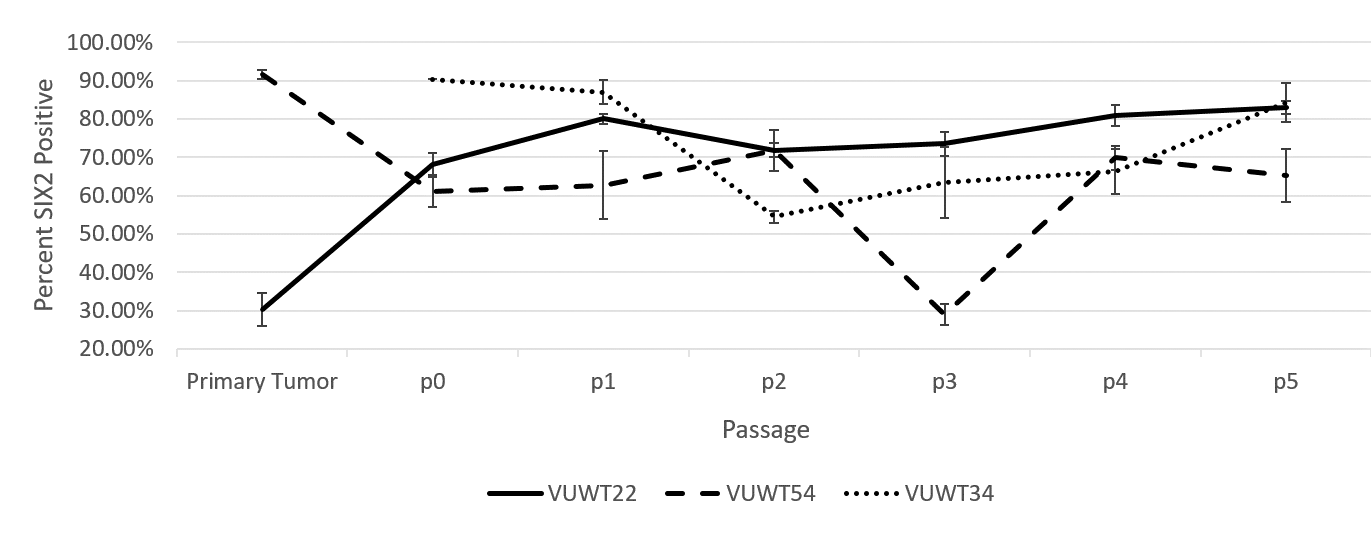

Supplement: Supplementary Figure 2 — A composite graph of flow cytometric analyses of SIX2 expression in all passages of cultured VUWT22, VUWT34, and VUWT54 cells. [file Image_2.tif]

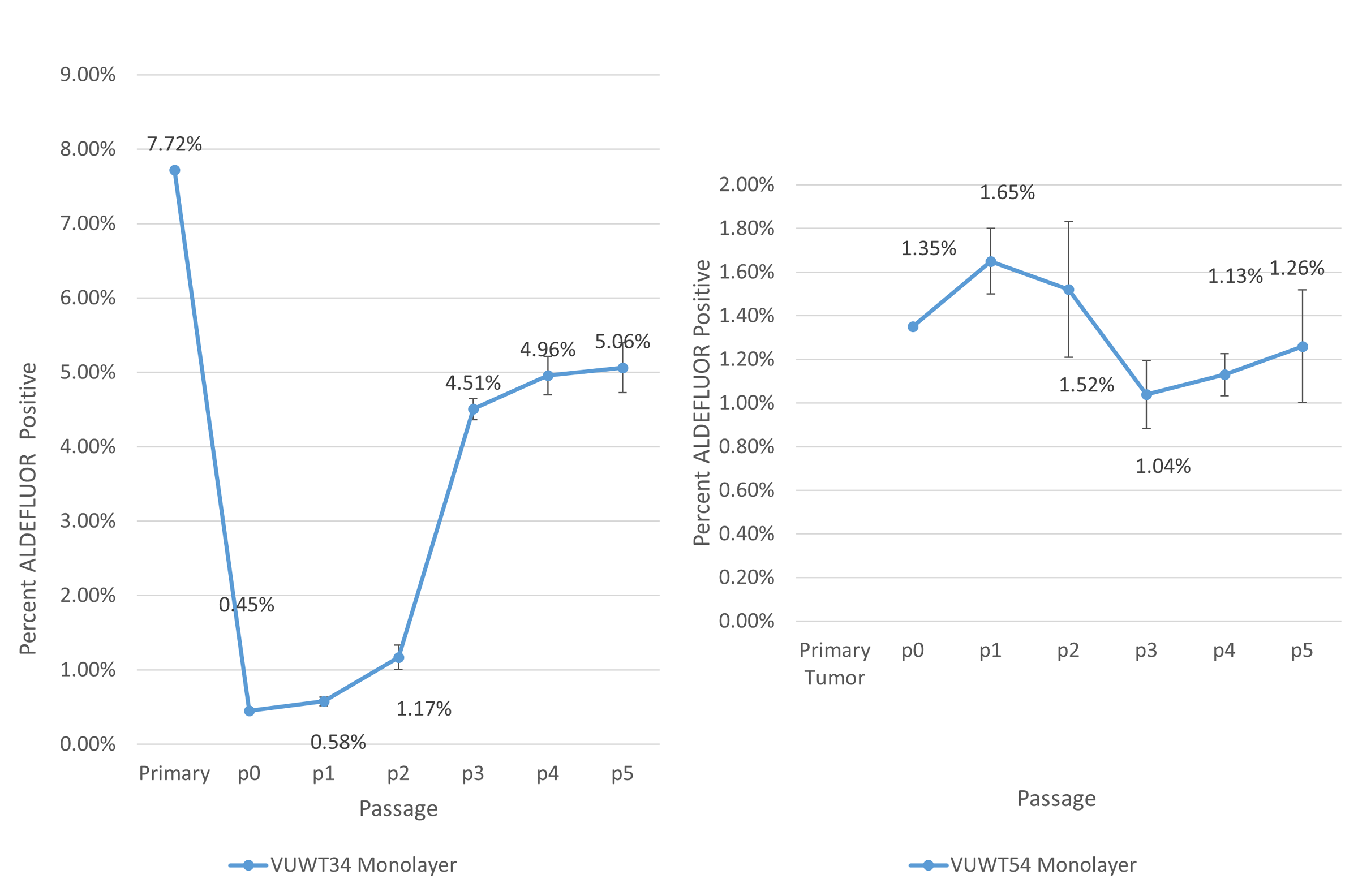

Supplement: Supplementary Figure 3 — Analysis of ALDH1+ cells for cultured passages of VUWT34 and VUWT54 cells using flow cytometry. [file Image_3.tif]

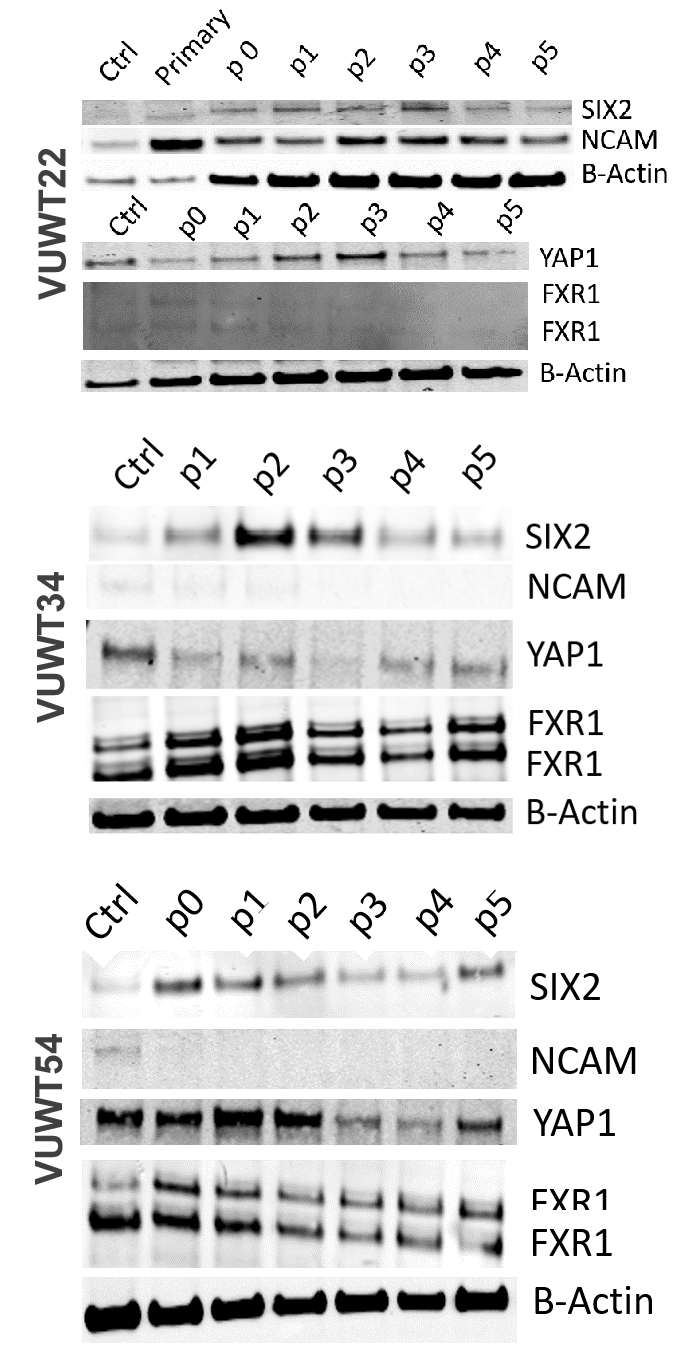

Supplement: Supplementary Figure 4 — Immunoblot analyses for Wilms tumor markers in cultured passages of VUWT22, VUWT34, and VUWT54 cells. [file Image_4.tif]

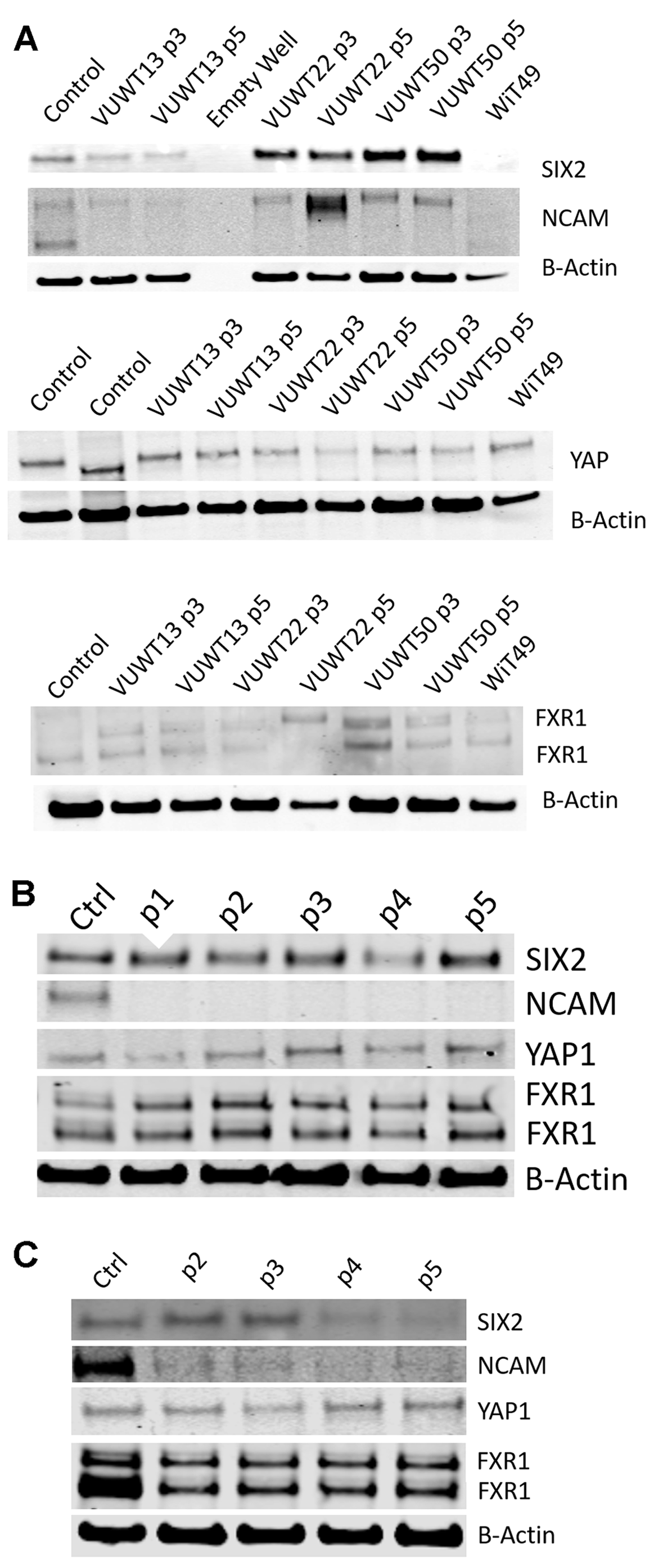

Supplement: Supplementary Figure 5 — Immunoblot analyses for Wilms tumor markers in spheroids from various cultured passages of; A - VUWT22, B - VUWT34, and C - VUWT54 cells. VUWT13 and VUWT50 were taken from these blots and included in Figure 6. HEK293 and embryonic mouse kidney were used as controls. [file Image_5.tif]
